# Water Dynamics in Fish Collagen Gels—Insight from NMR Relaxometry

**DOI:** 10.3390/ma17174438

**Published:** 2024-09-09

**Authors:** Maciej Osuch, Joanna Nowosad, Dariusz Kucharczyk, Michał K. Łuczyński, Adrianna Mieloch, Janusz Godlewski, Danuta Kruk

**Affiliations:** 1 Department of Physics and Biophysics, University of Warmia and Mazury in Olsztyn, Oczapowskiego 4, 10-719 Olsztyn, Poland; maciej.osuch@uwm.edu.pl; 2 Department of Research and Development, Chemprof, 11-041 Olsztyn, Poland; nowosad.joanna@gmail.com (J.N.); darekk56@gmail.com (D.K.); michal.luczynski@chemprof.pl (M.K.Ł.); 3 Department of Human Histology and Embryology, Medicine University of Warmia and Mazury in Olsztyn, Warszawska 30, 10-082 Olsztyn, Poland; adrianna.mieloch@uwm.edu.pl (A.M.); janusz.godlewski@uwm.edu.pl (J.G.)

**Keywords:** fish collagen, gels, dynamics, diffusion, NMR, relaxation

## Abstract

^1^H spin–lattice relaxation experiments have been performed for gels based on fish collagen in order to analyze water dynamics. The covered frequency range ranges from 10 kHz to 10 MHz; in some cases, the temperature has varied as well. The relaxation data have been reproduced in terms of two models of water motion—a model including two relaxation contributions associated with the diffusion of water molecules on the macromolecular surfaces and a second model being just a phenomenological power law. The concept of surface diffusion has led to a very good agreement with the experimental data and a consistent set of parameters, with the diffusion coefficients being about five orders of magnitude slower compared to bulk water for one of the pools and considerably faster for the second one (smaller by factors between 2 and 20 compared to bulk water). In some cases, the attempt to reproduce the data in terms of a power law has led to a good agreement with the experimental data (the power law factor varying between 0.41 and 0.57); however, in other cases, the discrepancies are significant. This outcome favors the concept of surface diffusion.

## 1. Introduction

Biomaterials are widely used in regenerative and esthetical medicine. A well-known example of such materials is dermal fillers (often based on hyaluronic acid), which are used to improve the appearance of skin and restore tissue volume. In regenerative medicine, biomaterials are used to support cell growth and, thus, tissue regeneration. The outcome of medical treatments depends largely on the molecular properties of the biomaterials used. By molecular properties, one understands not only the chemical composition but also the arrangement of different molecular fractions that determine the dynamical properties of the system. For instance, the arrangement of the macromolecular network determines the dynamics of the water molecules that form several pools, including fractions, of which the dynamics are strongly affected by constraints imposed by the macromolecular network and fractions that perform motion similar to that of bulk water. The structural and dynamical properties of biomaterials determine their macroscopic performance and their usefulness for medical applications. This implies that revealing the dynamical properties of biomaterials on the molecular level and understanding the relationship between the dynamical scenario and their functional properties is key to tailoring such materials for specific purposes. Fish collagen is considered a promising material for medical applications [1,2,3,4,5]. It is obtained from the skin or scales of fish (considered otherwise as waste), has antimicrobial properties, rarely leads to allergic reactions, and is environmentally sustainable. 

Nuclear Magnetic Resonance (NMR) relaxometry is a highly valuable method exploited in molecular science for investigating dynamical properties of systems of various complexity, from molecular and ionic liquids [6,7,8], via macromolecular systems (such as polymers or proteins) [9,10,11,12,13,14,15,16,17] to biomaterials [18] and tissues [19,20,21]. In contrast to “classical” NMR relaxation experiments performed at a single magnetic field (resonance frequency), in NMR relaxometry, the resonance frequency varies in a broad range, covering at least three orders of magnitude (from 10 kHz to 10 MHz for ^1^H). ^1^H relaxation rates (we shall focus on the spin–lattice relaxation process) depend on three main factors: the amplitude of ^1^H–^1^H dipole–dipole interactions (assuming there are no other NMR active nuclei with considerable gyromagnetic factors and abundances in the system) that depend on the molecular structure and arrangement, the time scale of the motion leading to time fluctuations of the dipole–dipole interactions (the time scale is described by a characteristic time constant, referred to as a correlation time), and the mechanism of the motion. With respect to the correlation time, the intrinsic property of spin–lattice relaxation processes is that at a given resonance, the most efficient relaxation pathway is associated with a motion occurring on a time scale matching the reciprocal resonance frequency (provided the amplitudes of dipole–dipole interactions corresponding to these relaxation pathways are comparable). This implies that by varying the resonance frequency, one can probe dynamical processes occurring on much different time scales in a single experiment. Moreover, NMR relaxometry offers the unique advantage of identifying the mechanism of the motion—one can differentiate, for instance, between translation diffusion, rotation motion (molecular tumbling), or specific types of polymer dynamics [18,22,23,24,25]. Following this line, one can distinguish between isotropic and anisotropic diffusion and sub-diffusive processes. To be even more specific, one is able to differentiate between three-dimensional (3D) isotropic translation movement (like in bulk liquids) and restricted, two-dimensional (2D) diffusion on macromolecular surfaces [25,26,27,28]. This unique potential of NMR relaxometry stems from the fact that relaxation rates are expressed as linear combinations of spectral density functions being Fourier transforms of the corresponding time correlation functions characterizing the motion that causes the fluctuations of the dipole–dipole interactions being the origin of the relaxation process. The mathematical form of the correlation function (and, hence, the spectral density) depends on the mechanism of the motion, and the shape of the frequency dependence of the spin–lattice relaxation rate is a fingerprint of the mechanism of the dynamical process. 

Profiting from this unique potential of NMR relaxometry, this work gives insight into the dynamics of water molecules in hydrogels based on fish collagen. The mechanism of the motion has been revealed and quantitatively described. Fish collagen is a promising material for regenerative medicine due to its biocompatibility, biodegradability, and structural similarity to human collagen.

## 2. Materials and Methods

### 2.1. Samples Preparation

African catfish (*Clarias gariepinus*), reared from egg to commercial fish under controlled conditions, were used for the study. Reproduction, fertilization, and incubation of eggs under controlled conditions were conducted in accordance with the methodology described in refs. [29,30]. The larvae were raised to the juvenile stage in recirculating aquaculture systems (RAS) at the Department of Ichthyology and Aquaculture, UWM in Olsztyn, according to the methodology described in ref. [31]. Commercial fish were reared using the methodology described in ref. [32]. During the entire rearing, the water temperature was maintained at 25 ± 0.1 °C (OxyGuard Pacific, Farum, Denmark). pH (Hanna HI 98128, Eden Way, Leighton Buzzard, UK), oxygenation, saturation (OxyGuard Pacific, Farum, Denmark), the level of nitrates (Hach LCK339, Ames, IA, USA), nitrites (Hach LCK341, Ames, IA, USA), and ammonia (Hach LCK303, Ames, IA, USA) [33]. During the final fattening period, the ammonia level was carefully monitored [34], as too high a level in the water may cause damage to the skin of African catfish. 

The skins were obtained from sexually mature individuals (*n* = 5; mean weight with SD: 1.98 ± 0.13 kg) of the farmed African catfish rearing with recirculation aquaculture systems (RAS). After the fish were killed using an overdose of MS-222 (Sigma–Aldrich, Saint Louis, MO, USA), the skins were separated from the fish bodies. Then, the skins were washed in cold water (7.0–9.0 °C), removed mucus, blood, and remnants of subcutaneous muscle tissue and placed in a refrigerator (7.0 ± 1.0 °C; Sharp, Osaka, Japan) until used. After 18 h, the skins were subjected to a disinfection and bleaching process using 0.5% hydrogen peroxide (Chempur, Piekary Śląskie, Poland) in a ratio of 1:10 (sample: solution). The bleaching process lasted for a period of 30 h. After this time, the skins were washed out again in cold water. Then cut the skin into small pieces (0.2 to 0.5 cm^2^). Collagen gel extraction was performed using 1% (samples 2 and 3) and 1.5% (samples 1, 4, 5, 6, 7) citric acid (Warchem, Warsaw, Poland) for 6 days. In the case of samples 2, 4, 6, and 7, skin without the pigmentation layer (epidermis) was used for extraction. The ratio of tissue to solution was 1:13 (sample 1) and 1:16 (samples 2 and 3). The extraction process was performed under refrigerated conditions (4.0 ± 1.0 °C; Sharp, Osaka, Japan). Sample 7 was subjected to a freeze-drying process for 20 h (Christ Gefriertrocknungsanlagen Freeze Dryers, Alpha 2-4 LDplus; Germany). The sample labeling is summarized in Table 1.

### 2.2. NMR Relaxometry Experiments

**Figure A1:**
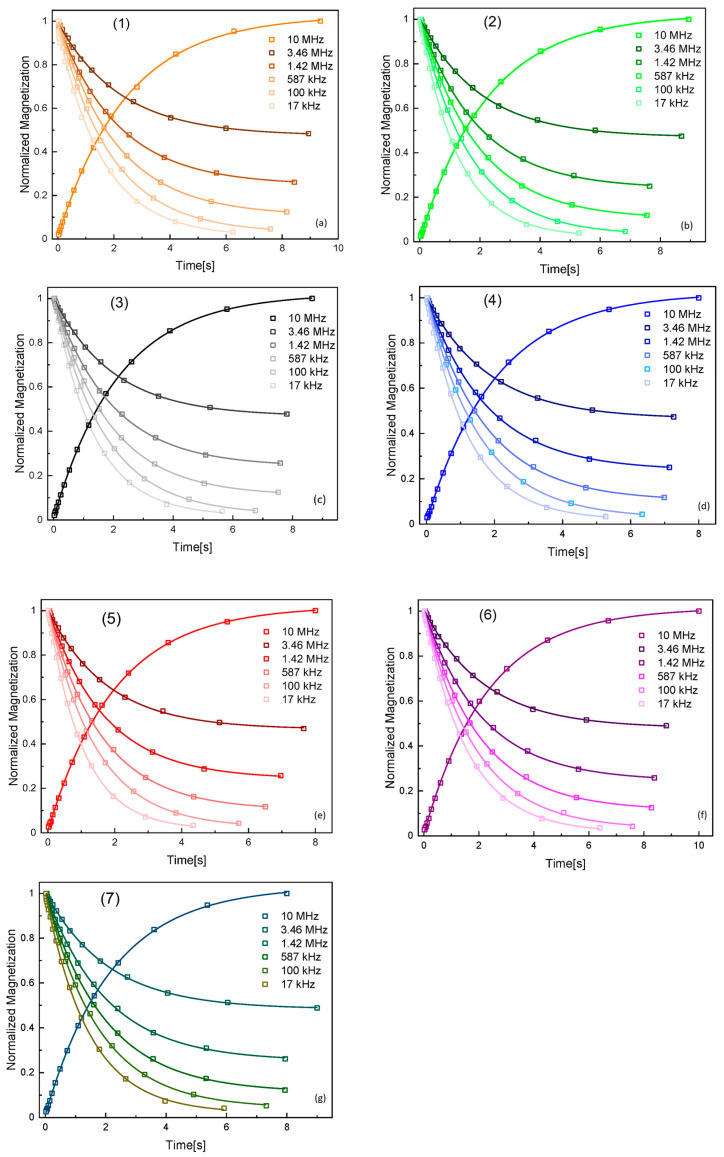
^1^H magnetization curves for samples 1–7 (**a**–**g**) at 298 K for selected frequencies. Solid lines—single exponential fits.

^1^H NMR relaxometry experiments were performed using SMARtracer FFC relaxometer (Stelar, Mede, Italy) in the frequency range from 10 kHz to 10 MHz. The temperature was stabilized using a built-in variable temperature controller (VTC) unit with an accuracy of 1 K. The relaxation process turned out to be single exponential for all samples, at all temperatures, and in the whole frequency range. Examples of magnetization curves (^1^H magnetization versus time) reproduced in terms of a single exponential function are shown in Appendix A (Figure A1). For complex systems including several molecular fractions one can expect bi-exponential (non-exponential) relaxation processes. Ensuring that the relaxation process is single exponential is crucial for the strategy of the data analysis. The observed exponentiality confirms that the observed relaxation process is associated with water molecules.

### 2.3. Theory

^1^H relaxation processes are caused by ^1^H–^1^H magnetic dipole–dipole interactions. The spin–lattice relaxation rate, $R_{1}\left(\omega \right)$ (ω denotes ^1^H resonance frequency in angular frequency units), is given as follows (Equation (1)) [35,36]:

(1) $$R_{1}\left(\omega \right)=C_{DD}\left[J\left(\omega \right)+4J\left(2\omega \right)\right]$$

The quantity $J\left(\omega \right)$ is referred to as a spectral density function. The spectral density is defined as a Fourier transform of the corresponding time correlation function describing stochastic fluctuations of the dipole–dipole interactions causing the relaxation process. The parameter $C_{DD}$ denotes a dipolar relaxation constant. As pointed out in the Introduction, the mathematical form of the spectral density function depends on the mechanism of the molecular motion. One can expect that for complex systems, including molecular fractions undergoing different kinds of motion, the overall relaxation rate includes contributions associated with different pools of ^1^H nuclei and different mechanisms of motion. One of the anticipated mechanisms of motion is the translation diffusion of water molecules on the surface of the macromolecules (referred to as two-dimensional (2D) translation diffusion). The spectral density function characterizing this kind of motion, $J_{2D}^{trans}\left(\omega \right)$, is given as follows (Equation (2)) [18,24,27,28]:

(2) $$J_{2D}^{trans}\left(\omega \right)=\tau_{trans}\ln\left[\frac{1+{\left(\omega \tau_{trans}\right)}^{2}}{{\left(\frac{\tau_{trans}}{\tau_{res}}\right)}^{2}+{\left(\omega \tau_{trans}\right)}^{2}}\right]$$

The correlation time $\tau_{trans}$ is defined as follows: $\tau_{trans}=\frac{d^{2}}{2D_{trans}}$, where $D_{trans}$ denotes the diffusion coefficient, d is the diameter of the water molecule, $\tau_{res}$ denotes the residence lifetime of water molecules on the surface (the diffusion of water molecules near the macromolecule surface is interrupted by forming temporary hydrogen bonds with the protein). One should note that for long residence lifetimes, Equation (2) converges to a simpler form (Equation (3)):

(3) $$J_{2D}^{trans}\left(\omega \right)=\tau_{trans}\ln\left[\;1+{\left(\omega \tau_{trans}\right)}^{-2}\right]$$

Anticipating the result, the ^1^H spin–lattice relaxation data can be reproduced as a sum of the following terms (Equation (4)):

(4) $$\begin{array}{ll} R_{1}\left(\omega \right) & =R_{1,\;slow}^{trans}\left(\omega \right)+R_{1,\;fast}^{trans}\left(\omega \right)+A\\ & =C_{DD}^{slow}\tau_{trans}^{slow}\left[ln\left[1+{\left(\omega \tau_{trans}^{slow}\right)}^{-2}\right]+4ln\left[1+{\left(2\omega \tau_{trans}^{slow}\right)}^{-2}\right]\right]\\ & \;+C_{DD}^{fast}\tau_{trans}^{fast}\left[ln\left[1+{\left(\omega \tau_{trans}^{fast}\right)}^{-2}\right]+4ln\left[1+{\left(2\omega \tau_{trans}^{fast}\right)}^{-2}\right]\right]+A \end{array}$$

The first relaxation term, $R_{1,\;slow}^{trans}\left(\omega \right)$, describes the relaxation contribution associated with surface diffusion of water molecules—it includes the parameters $\tau_{trans}^{slow}$ and $C_{DD}^{slow}$, representing the translation correlation time and the corresponding dipolar relaxation constant. The index “*slow*” becomes clear when one looks at the second relaxation contribution, $R_{1,\;fast}^{trans}\left(\omega \right)$. This relaxation term also describes two-dimensional surface diffusion, but occurring on a shorter time scale (being faster) compared to the diffusion process attributed to the $R_{1,\;slow}^{trans}\left(\omega \right)$ term; $\tau_{trans}^{fast}$ and $C_{DD}^{fast}$ describe the corresponding correlation time and the dipolar relaxation constant. Eventually, the frequency independent term, A, represents a relaxation contribution associated with bulk (free) water fraction. 

There is an alternative to the model of Equation (4), expressed as follows (Equation (5)):

(5) $$R_{1}\left(\omega \right)=R_{1}^{power-law}\left(\omega \right)+A=C\omega^{-\beta }+A$$

The power law frequency dependence of the relaxation contribution can be attributed to the dynamics of the protein backbones probed indirectly via the relaxation process of bound water molecules [12,13,14,18,33,37]. The outcome of the comparison of these two models (Equations (4) and (5)) is presented in Results.

## 3. Results 

**Figure 1:**
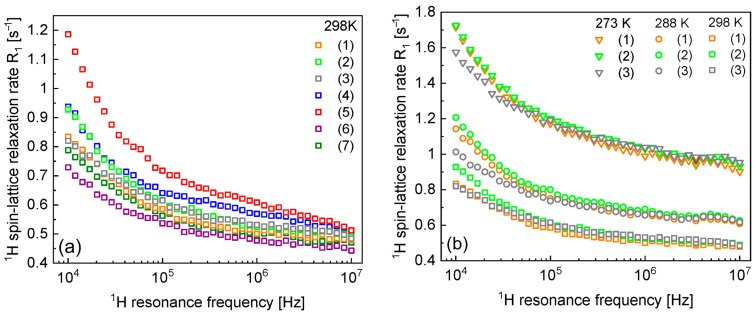
(**a**) ^1^H spin–lattice relaxation data for samples 1–7 at 298 K and (**b**) ^1^H spin–lattice relaxation data for samples 1–3 at 298 K, 288 K, and 273 K.

Figure 1a shows the ^1^H spin–lattice relaxation data obtained at 298 K for the seven samples listed in Table 1. Figure 1b shows how the relaxation rates for samples 1–3. change with temperature. Temperature-induced differences in relaxation rates are observed at low magnetic fields.

**Figure A2:**
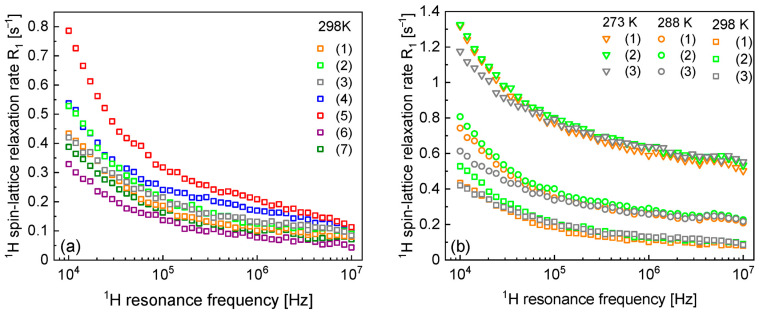
(**a**) ^1^H spin–lattice relaxation data for samples 1–7 at 298 K after subtracting a relaxation contribution attributed to bulk water (0.4 s^−1^); (**b**) ^1^H spin–lattice relaxation data for samples 1–3 at 298 K, 288 K, and 273 K after subtracting a relaxation contribution attributed to bulk water (0.4 s^−1^).

Before proceeding with the data analysis, the relaxation contribution associated with bulk (free) water (0.4 s^−1^) has been subtracted from the overall relaxation rates. The outcome is shown in Appendix A (Figure A2) to reveal the relaxation process associated with the fraction of water molecules whose dynamics are influenced by interactions with the macromolecular fraction.

**Figure 2:**
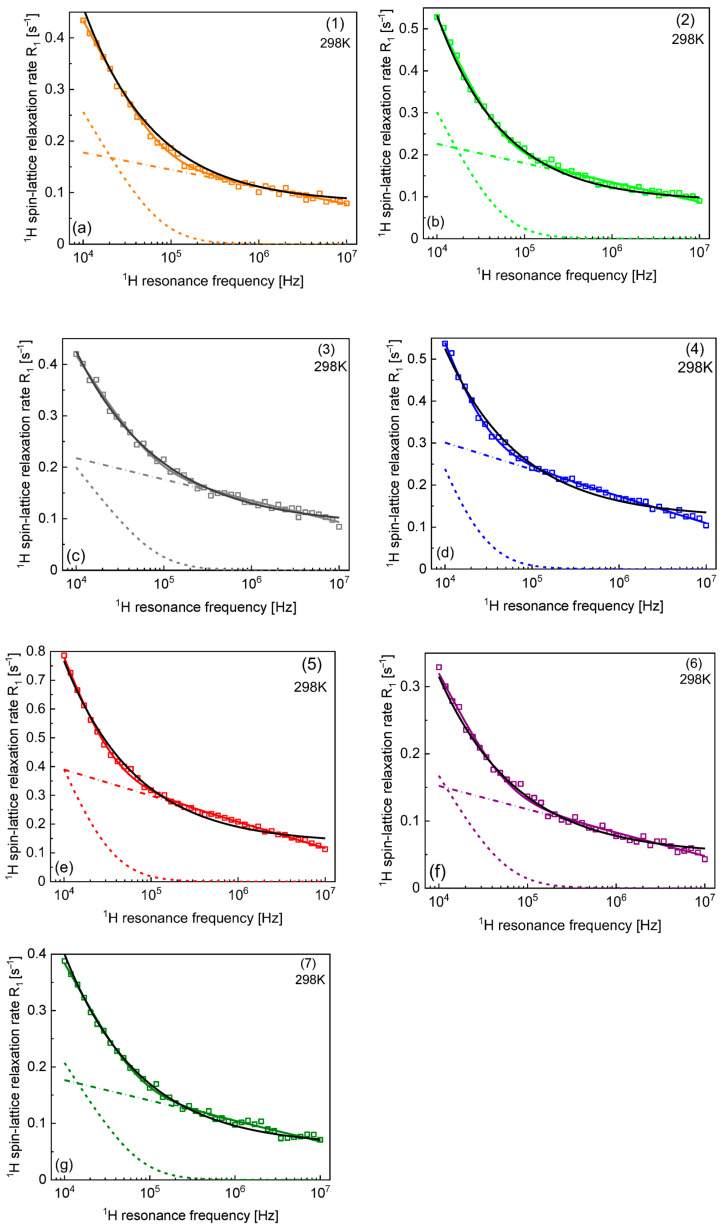
^1^H spin–lattice relaxation rates for samples 1–7 (from (**a**) to (**g**)) at 298 K. Solid colour lines—fit of the overall relaxation rate in terms of Equation (4) decomposed into $R_{1,\;slow}^{trans}$ (dashed colour lines) and $R_{1,\;fast}^{trans}$ (dash-dotted colour line). Solid black lines—fits in terms of Equation (5).

We begin the analysis with the data collected for samples 1–7 at 298 K. Figure 2 shows the outcome of the analysis by means of the models of Equations (4) and (5). The relaxation contribution associated with the free water fraction (0.4 s^−1^) has been subtracted from the overall relaxation rates to make the details of the analysis more visible.

**Figure 3:**
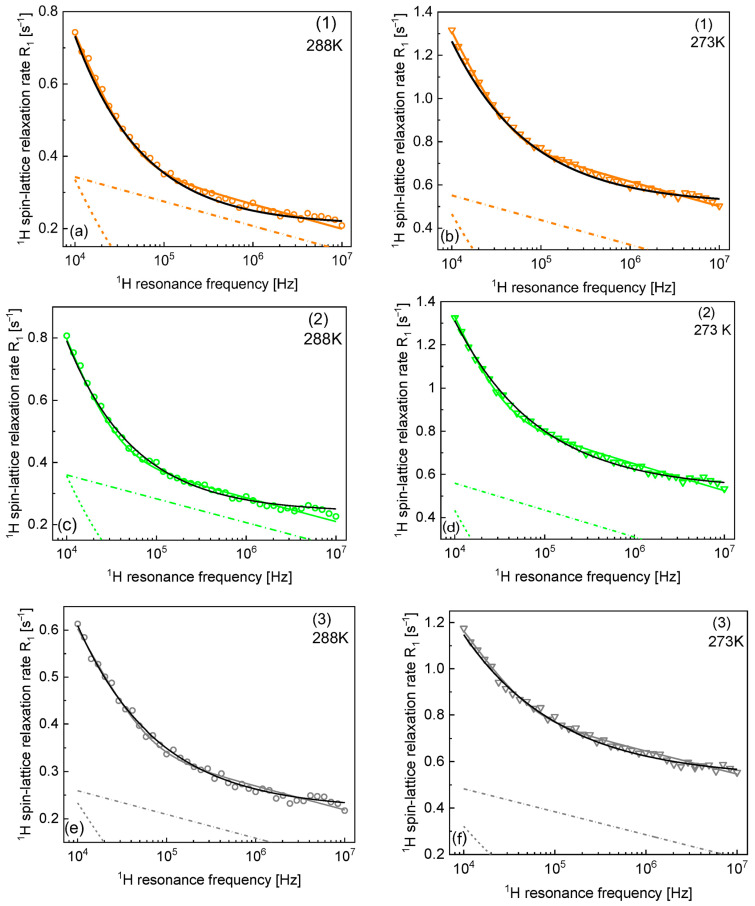
^1^H spin–lattice relaxation rates for samples 1–3 at 288 K (**a**,**c**,**e**) and 273 K (**b**,**d**,**f**). Solid colour lines—fit of the overall relaxation rate in terms of Equation (4) decomposed into $R_{1,\;slow}^{trans}$ (dashed colour lines) and $R_{1,\;fast}^{trans}$ (dash-dotted colour line). Solid black lines—fits in terms of Equation (5).

The parameters obtained from the analysis in terms of Equation (4) are collected in Table 2, while the parameters obtained by using Equation (5) are included in Table 3. The table also includes the parameters obtained for samples 1–3 at 288 K and 273 K. The results of the analysis of the data collected at the lower temperatures (288 K and 273 K) are shown in Figure 3. Again, the relaxation contribution associated with the free water fraction (0.4 s^−1^) at 298 K has been subtracted from the overall relaxation rates. The value of 0.4 s^−1^ has been added to the frequency-independent term, A, reported in Table 2 and Table 3. ^1^H spin–lattice relaxation rates for samples 1–3 at 288 K and 273 K are shown in Figure 3.

## 4. Discussion

The characteristic feature of the ^1^H spin–lattice relaxation rates obtained for all samples is their linear dependence on the logarithm of the resonance frequency at lower frequencies. This observation suggests translation dynamics of water molecules on the surface of the macromolecular fraction as the mechanism of motion associated with the relaxation process. Consequently, we have proposed the model of Equation (4) assuming the presence of two pools of water molecules performing translation diffusion in the vicinity of the macromolecular surface (in addition to a pool of water molecules not affected by the presence of the macromolecular fraction; such pools of water molecules are often referred to as “free” water). The relaxation rate of the “free” water fraction, represented by the frequency-independent term, A, has been estimated as 0.4 s^−1^ at 298 K (that corresponds to the relaxation rate of bulk water); the value has been confirmed by fitting the relaxation data for samples 1 and 2 at 298 K. Then, for samples 3–7 at 298 K, A = 0.4 s^−1^ has been set. One should point out at this stage that we have tested models including, in addition to a relaxation contribution associated with surface diffusion, a contribution expressed in terms of Lorentzian spectral densities [11,25] and isotropic (three dimensional) translation diffusion [30], concluding that the model of Equation (4) including as a second relaxation contribution a term also representing surface (two dimensional) diffusion. Focusing on the results of the analysis for 298 K (Table 2) one can say that the dipolar relaxation constant $C_{DD}^{slow}$ varies between 1.13 × 10^4^ Hz^2^ for sample 5 and 5.84 × 10^3^ Hz^2^ for sample 6. The difference does not exceed factor 2 and can be related to both effects—differences in the populations of the water fraction undergoing the slow translation movement or/and somewhat different exchange dynamics between pools of hydrogen atoms. It is worth reminding that for sample 6, skin without the pigmentation layer is used in contrast to sample 5. Independently of the origin, the effect is small. The same can be said about the corresponding correlation time, $\tau_{trans}^{slow}$, that is of the order of microseconds and varies between 1.13 × 10^−6^ s for sample 3 and 3.60 × 10^−6^ s for sample 4, rendering the corresponding translation diffusion coefficient, $D_{trans}^{slow}$ ranging from 3.59 × 10^−14^ m^2^/s to 1.09 × 10^−14^ m^2^/s. One should note that the diffusion is by five orders of magnitude slower compared to water diffusion in bulk (about 2 × 10^−9^ m^2^/s). As far as the relaxation contribution associated with the faster diffusion is concerned, the dipolar relaxation constant $C_{DD}^{fast}$ ranges between 3.91 × 10^6^ Hz^2^ for sample 6 and 3.65 × 10^7^ Hz^2^ for sample 1, that encompasses one order of magnitude and can be treated as indication of the population of the corresponding water fraction in the samples (the dipolar relaxation constants are proportional to the mole fractions of water molecules in the water pools). Again, for sample 6, skin without the pigmentation layer was used in contrast to sample 1. Differences in the correlation time $\tau_{trans}^{fast}$ (and, hence, the translation diffusion coefficient, $D_{trans}^{fast}$) are also significant—the values range from 3.94 × 10^−11^ s for sample 1 to 4.81 × 10^−10^ s for sample 5, again spanning an order of magnitude. The corresponding diffusion coefficient varies between 1.03 × 10^−9^ m^2^/s for sample 1 and 8.44 × 10^−11^ m^2^/s for sample 5. It is important to notice that the larger diffusion coefficient differs only by factor 2 from that for bulk water. In both cases (sample 1 and sample 5), the extraction procedure was the same, but for sample 1, the tissue-to-solution ratio is 1:16, while for sample 5 it yields 1:18. With decreasing temperature the dipolar relaxation constant $C_{DD}^{fast}$ remains unchanged, while the translation dynamics slows down by a factor of about 3. As far as $D_{trans}^{slow}$ is concerned, the motion slows down by about factor 2, while the dipolar relaxation constant $C_{DD}^{slow}$ somewhat increases at 273 K, likely due to reduced exchange processes. The term A increases with decreasing temperature as expected (the dynamics of bulk water slows down with decreasing temperature, leading to an increase of the relaxation rate).

Despite the consistency of the description, one should be aware that the model includes four parameters (not including the term A) used to reproduce relaxation data that show relatively weak frequency dependence (the relaxation rates in the low and high-frequency limits differ by factor 2–3). This implies that one should address the subject of unambiguity of the interpretation. Therefore, we have attempted to reproduce the data in terms of Equation (5). It has turned out that the relaxation data can be reproduced, to some extent, in terms of the power law $C\omega^{-\beta }$ and the term A varying between 0.45 s^−1^ and 0.57 s^−1^ for 298 K (being close to the relaxation rate of bulk water) and reaching higher values with decreasing temperature (Table 2). The quality of the fits is worse than those based on Equation (4). In some cases (sample 2 at 298 K), the agreements with the experimental data are, in fact, comparable for both models; however, for other cases (sample 4 or sample 5 at 298 K), the power law model does not capture the shape of the frequency dependence of the relaxation rates. For samples 1–3, relatively good agreement with the experimental data is also observed at 288 K and 273 K, with similar power law factors. The power law concept represents a phenomenological approach and does not provide any information about the time scale of the molecular motion. The fact that, in some cases, it approximates the data relatively well does not mean that the model of Equation (4) is not justified. Nevertheless, one should be aware of this finding.

## 5. Conclusions

The thorough analysis of the relaxation data indicates the presence of two water pools (in addition to the third one referred to as free water). Water molecules belonging to those pools perform two-dimensional diffusions on the macromolecular surfaces. The difference between the dynamics of both pools lies in the time scale of the diffusion process—for one of these pools, the diffusion coefficient is five orders of magnitude slower compared to bulk water, while for the second one, the diffusion is much faster: the diffusion coefficient ranges from values about 20 times smaller than the diffusion coefficient for bulk water to values smaller only by a factor of about 2. The partially successful attempt to reproduce the relaxation data in terms of a power law underlines the need for a cautious analysis aimed at reaching consistency for all cases considered, including changes in the relaxation properties caused by temperature.

It would be of interest, in further studies, to look for relationships between these findings and water activity. The role and manner of the influence of free water (not bound) in seafood and collagen prepared for biomedical purposes are definitely different. In food processing, free water often ranges from 5 to 95%, and its percentage depends on the type of raw material and the method of its preservation and storage. Often, excess free water can contribute to the processes of reducing the quality of raw seafood or even its damage. The issue of the origin of free water in food, as well as its chemical and microbiological purity, is also important. In these studies, free water is free from chemical and microbiological contamination. Its role is also different—a small amount of it protects samples in the laboratory from drying out during contact with air when opening containers with samples and during the tests.

## Figures and Tables

**Table 1:** Labeling and description of the samples.

Sample Label	Description
1	collagen gel extraction with 1.5% citric acid; tissue-to-solution ratio: 1:13
2	collagen gel extraction with 1% citric acid; skin without the pigmentation layer used; tissue-to-solution ratio: 1:16
3	collagen gel extraction with 1% citric acid; tissue-to-solution ratio: 1:16
4	collagen gel extraction with 1.5% citric acid; skin without the pigmentation layer used; tissue-to-solution ratio: 1:18
5	collagen gel extraction with 1.5% citric acid; tissue-to-solution ratio: 1:18
6	collagen gel extraction with 1.5% citric acid; skin without the pigmentation layer used; tissue-to-solution ratio: 1:18
7	collagen gel extraction with 1.5% citric acid; skin without the pigmentation layer used; subjected to freeze-drying process; tissue-to-solution ratio: 1:18

**Table 2:** Parameters obtained as a result of reproducing the ^1^H spin–lattice relaxation data in terms of Equation (4). The translation diffusion coefficients, $D_{trans}^{slow}$ and $D_{trans}^{fast}$, have been obtained from the following relationships: $\tau_{trans}^{slow}=\frac{d^{2}}{2D_{trans}^{slow}}$ and $\tau_{trans}^{fast}=\frac{d^{2}}{2D_{trans}^{fast}}$, where d denotes the diameter of water molecule, d = 2.85 Å.

Sample	Temp. [K]	$C_{DD}^{slow}$ [Hz^2^]	$\tau_{trans}^{slow}$ [s]	$C_{DD}^{fast}$ [Hz^2^]	$\tau_{trans}^{fast}$ [s]	A [s^−1^]	$D_{trans}^{slow}$ [m^2^/s]	$D_{trans}^{fast}$ [m^2^/s]
1	298	(1.04 ± 0.05) × 10^4^	(1.22 ± 0.09) × 10^−6^	(3.65 ± 1.53) × 10^7^	(3.94 ± 1.90) × 10^−11^	0.40 ± 0.01	3.33 × 10^−14^	1.03 × 10^−9^
1	288	(1.04 ± 0.05) × 10^4^	(2.25 ± 0.35) × 10^−6^	(3.65 ± 1.53) × 10^7^	(8.07 ± 0.22) × 10^−11^	0.46 ± 0.02	1.81 × 10^−14^	4.56 × 10^−10^
1	273	(1.42 ± 0.26) × 10^4^	(2.36 ± 0.23) × 10^−6^	(3.65 ± 1.53) × 10^7^	(1.36 ± 0.06) × 10^−10^	0.69 ± 0.01	1.72 × 10^−14^	2.99 × 10^−10^
2	298	(1.03 ± 0.05) × 10^4^	(1.75 ± 0.14) × 10^−6^	(1.80 ± 0.58) × 10^7^	(1.11 ± 0.04) × 10^−10^	0.40 ± 0.01	2.32 × 10^−14^	3.66 × 10^−10^
2	288	(1.03 ± 0.05) × 10^4^	(2.84 ± 0.22) × 10^−6^	(1.80 ± 0.58) × 10^7^	(1.85 ± 0.05) × 10^−10^	0.48 ± 0.02	1.42 × 10^−14^	2.20 × 10^−10^
2	273	(1.23 ± 0.21) × 10^4^	(3.04 ± 0.39) × 10^−6^	(1.80 ± 0.58) × 10^7^	(3.03 ± 0.12) × 10^−10^	0.76 ± 0.01	1.34 × 10^−14^	1.34 × 10^−10^
3	298	(8.42 ± 0.17) × 10^3^	(1.13 ± 0.13) × 10^−6^	(3.58 ± 1.47) × 10^7^	(5.02 ± 0.24) × 10^−11^	0.40	3.59 × 10^−14^	8.09 × 10^−10^
3	288	(8.42 ± 0.17) × 10^3^	(1.55 ± 0.10) × 10^−6^	(3.58 ± 1.47) × 10^7^	(6.23 ± 0.22) × 10^−11^	0.51 ± 0.01	2.62 × 10^−14^	6.52 × 10^−10^
3	273	(1.08 ± 0.09) × 10^4^	(1.82 ± 0.21) × 10^−6^	(3.58 ± 1.47) × 10^7^	(1.20 ± 0.07) × 10^−10^	0.76 ± 0.01	2.23 × 10^−14^	3.38 × 10^−10^
4	298	(6.56 ± 0.18) × 10^3^	(3.60 ± 0.35) × 10^−6^	(1.57 ± 0.25) × 10^7^	(1.78 ± 0.35) × 10^−10^	0.40	1.13 × 10^−14^	2.28 × 10^−10^
5	298	(1.13 ± 0.26) × 10^4^	(2.82 ± 0.18) × 10^−6^	(8.22 ± 0.91) × 10^6^	(4.81 ± 0.65) × 10^−10^	0.40	1.44 × 10^−14^	8.44 × 10^−11^
6	298	(5.84 ± 0.04) × 10^3^	(1.67 ± 0.21) × 10^−6^	(3.91 ± 0.11) × 10^6^	(3.87 ±1.35) × 10^−10^	0.40	2.43 × 10^−14^	1.05 × 10^−10^
7	298	(8.26 ± 0.06) × 10^3^	(1.26 ± 0.12) × 10^−6^	(1.31 ± 0.34) × 10^7^	(1.20 ± 0.48) × 10^−10^	0.40	3.22 × 10^−14^	3.38 × 10^−10^

**Table 3:** Parameters obtained as a result of reproducing the ^1^H spin–lattice relaxation data in terms of Equation (5). We do not provide units for C as they depend on β due to the phenomenological nature of the model.

Sample	Temp. [K]	C	β	A [s^−1^]
1	298	54.9 ± 7.7	0.54 ± 0.02	0.48 ± 0.02
1	288	90.9 ± 8.2	0.56 ± 0.01	0.61 ± 0.01
1	273	68.9 ± 10.4	0.49 ± 0.01	0.81 ± 0.01
2	298	84.4 ± 10.0	0.57 ± 0.01	0.49 ± 0.01
2	288	105.1 ± 15.4	0.57 ± 0.02	0.64 ± 0.01
2	273	54.1 ± 6.3	0.48 ± 0.01	0.91 ± 0.01
3	298	20.6 ± 3.1	0.45 ± 0.01	0.48 ± 0.01
3	288	32.5 ± 4.6	0.48 ± 0.02	0.62 ± 0.02
3	273	27.0 ± 3.9	0.41 ± 0.02	0.93 ± 0.02
4	298	40.8 ± 8.9	0.50 ± 0.03	0.52 ± 0.02
5	298	79.1 ± 11.9	0.52 ± 0.02	0.53 ± 0.01
6	298	24.20 ± 3.89	0.49 ± 0.02	0.45 ± 0.01
7	298	31.02 ± 4.07	0.49 ± 0.01	0.47 ± 0.02

## Data Availability

The data that support the findings are available from the corresponding author upon request.

## Appendix A

Examples of time evolution of ^1^H magnetisation (Figure A1); ^1^H spin–lattice relaxation rates after subtracting a relaxation contribution attributed to bulk water (Figure A2).
